# Plasma p-tau217, p-tau181 and Aβ42/40 for Alzheimer’s disease diagnosis: ROC accuracy and 18F-florbetapir amyloid PET-CT concordance

**DOI:** 10.17305/bb.2026.13556

**Published:** 2026-03-03

**Authors:** Zhengying Jing, Rong Wang, Chunmei Zhao, Jiawei Ma, Guisheng Chen

**Affiliations:** 1 The First Clinical Medical College, Ningxia Medical University, Yinchuan, China; 2 Department of Nuclear Medicine, General Hospital of Ningxia Medical University, Yinchuan, China; 3 Department of Neurology, General Hospital of Ningxia Medical University, Yinchuan, China; 4 Ningxia Medical University Key Laboratory of Cranial Brain Diseases, General Hospital of Ningxia Medical University, Yinchuan, China

**Keywords:** Alzheimer’s disease, plasma biomarkers, p-tau217, p-tau181, Aβ-42/40

## Abstract

Early diagnosis of Alzheimer’s disease (AD) presents significant challenges. This study assessed the diagnostic utility of seven plasma biomarkers and PET-CT imaging in cognitively healthy individuals (HC), those with mild cognitive impairment (MCI), and AD patients. Seventy participants (20 with MCI, 35 with AD, and 15 HC) underwent plasma testing for amyloid-beta 40 (Aβ40), amyloid-beta 42 (Aβ42), the Aβ42/Aβ40 ratio, phosphorylated tau 181 (p-tau181), p-tau217, glial fibrillary acidic protein (GFAP), and neurofilament light chain (NfL), along with cognitive assessments using the Mini-Mental State Examination (MMSE). Statistical comparisons among groups were performed, and receiver operating characteristic (ROC) curves were utilized to evaluate diagnostic accuracy. Spearman’s correlation coefficient was applied to examine the relationships between biomarkers and MMSE scores. Additionally, 18 patients, including 14 with AD and 4 with MCI, underwent 18F-Florbetapir (18F-AV45) PET-CT amyloid imaging. The consistency between plasma biomarkers and PET-CT in detecting amyloid pathology was evaluated using Cohen’s Kappa. Plasma Aβ42 levels and the Aβ42/Aβ40 ratio were significantly lower in AD patients compared to those with MCI and HC (*P <* 0.05), while levels of p-tau181, p-tau217, NfL, and GFAP were significantly elevated (*P <* 0.05). Aβ42 and the Aβ42/Aβ40 ratio exhibited positive correlations with MMSE scores (*P <* 0.01), whereas p-tau181, p-tau217, GFAP, and NfL demonstrated negative correlations (*P <* 0.001). The plasma Aβ42/Aβ40 ratio, p-tau181, and p-tau217 levels showed significant concordance with 18F-AV45 PET-CT results for detecting amyloid deposition (*P <* 0.05). A reduced plasma Aβ42/Aβ40 ratio, along with increased p-tau181 and p-tau217 levels, is significantly associated with a clinical diagnosis of AD, cognitive decline (as indicated by lower MMSE scores), and positive amyloid deposition on PET-CT. These three core biomarkers, when combined with GFAP and NfL, may enhance diagnostic accuracy for AD in cross-sectional assessments, particularly when integrated with imaging and cognitive evaluations.

## Introduction

Alzheimer’s disease (AD) is a progressive neurodegenerative disorder and the leading cause of dementia, responsible for 60% to 80% of all dementia cases [1]. It profoundly impacts the health and quality of life of millions of elderly individuals globally. As human lifespan increases, the incidence of AD rises proportionally, posing significant challenges to public health. A systematic review and meta-analysis from China further indicates that both the incidence and prevalence of AD are increasing [2].

AD is characterized as a continuous biological pathological process marked by the deposition of amyloid-beta (Aβ) and pathological tau proteins. This process begins years before the onset of clinical symptoms and is identified and staged *in vivo* using the AT(N) biomarker system [3]. The symptomatic stages of AD include mild cognitive impairment (MCI) and dementia (AD dementia). Once patients progress to the AD dementia stage, reversing the disease becomes increasingly challenging. Therefore, the management of AD dementia highlights the necessity of “early detection and early intervention” [4]. This is particularly important given that several disease-modifying therapies have shown efficacy in slowing progression during the early symptomatic or prodromal stages of AD [5]. The ongoing development of monoclonal antibody therapies and other innovative treatments underscores the critical need for early diagnosis of Alzheimer’s disease [6].

However, the clinical application of lecanemab and aducanumab presents significant limitations. Their efficacy is modest, resulting in a decline slowdown of approximately 27% over 18 months without halting disease progression. Substantial safety risks, particularly amyloid-related imaging abnormalities (ARIA), necessitate intensive magnetic resonance imaging (MRI) monitoring. Treatment is also limited to early-stage, amyloid-positive patients, requires intravenous administration, and incurs high costs, which restrict accessibility. The long-term effects of these therapies remain uncertain [7].

In recent years, advancements in detection techniques for biological markers of Alzheimer’s disease in plasma have provided more convenient and safer methods for clinical diagnosis. Among these, plasma biomarkers such as phosphorylated tau 181 (p-tau181), phosphorylated tau 217 (p-tau217), and total tau (t-tau) have shown promising applications in clinical research on AD. The Alzheimer’s Association, in its latest revised diagnostic and staging criteria released in 2024, has formally included blood-based biomarkers in the Core 1 diagnostic criteria for Alzheimer’s Disease [8]. This study investigated the relationship between plasma biomarkers, cognitive function, and cerebral amyloid deposition. Expression levels of Aβ40, Aβ42, the Aβ42/Aβ40 ratio, p-tau181, p-tau217, glial fibrillary acidic protein (GFAP), and neurofilament light chain (NfL) were measured in patients with mild cognitive impairment and Alzheimer’s disease. Cognitive function was assessed, and 18F-AV45 positron emission tomography–computed tomography (PET-CT) imaging was performed to evaluate β-amyloid deposition in the brain. Notably, unlike many studies relying on specialized platforms (e.g., Simoa), this study innovatively evaluated these core AD biomarkers using a fully automated chemiluminescence immunoassay. Overall, this research aimed to assess Alzheimer’s disease plasma biomarkers and their association with amyloid PET within a clinically accessible testing framework.

## Materials and methods

### Participants

This study consecutively enrolled eligible outpatients from the Department of Neurology at the General Hospital of Ningxia Medical University in Yinchuan between December 2024 and February 2025. All AD and MCI patients were recruited during visits initiated for diagnostic evaluations of cognitive complaints reported by themselves or their family members. The cohort included 20 patients diagnosed with MCI and 35 patients diagnosed with AD. Concurrently, 15 cognitively healthy controls (HC) were enrolled from individuals undergoing routine health examinations at a Health Management Center during the same period. The HC participants, who volunteered after being preliminarily matched to the patient groups based on age and gender, provided informed consent to participate.

All participants, including those in the AD and MCI groups and the HC group, underwent cognitive screening using the Mini-Mental State Examination (MMSE). Specifically, all patients in the AD group received diagnoses from at least one neurologist according to the criteria for “probable AD” established by the National Institute of Neurological and Communicative Disorders and Stroke and the Alzheimer’s Disease and Related Disorders Association (NINCDS-ADRDA) Work Group. Patients in the MCI group were diagnosed based on the 2018 Chinese Guidelines for the Diagnosis and Treatment of Dementia and Cognitive Disorders (Part V). Exclusion criteria for all participants included a family history of AD, any life-threatening systemic illness, or significant hearing or visual impairments that could compromise testing. Efforts were made to match the HC group to the patient groups concerning age, gender, and general living environment.

### Plasma biomarker testing

Five milliliters of venous blood were drawn from the cubital vein in a non-fasting state into ethylenediaminetetraacetic acid (EDTA) tubes. Hemolyzed samples were identified and excluded from analysis. The tubes were immediately inverted gently 8–10 times and centrifuged within 1 h (3000 r/min, 10 min) to ensure the absence of sediment or impurities. Two milliliters of the upper plasma were then transferred into reaction cups. All samples were tested fresh without undergoing freeze-thaw cycles, and analysis was performed immediately after centrifugation. The levels of Aβ40, Aβ42, the Aβ42/Aβ40 ratio, p-tau181, p-tau217, GFAP, and NfL were measured using a fully automated chemiluminescence immunoassay analyzer (Shine i2910, Shenzhen IncreCare Biotechnology Co., Ltd.) with reagent kits from Nanjing Vazyme Medical Technology Co., Ltd., employing the direct chemiluminescence immunoassay (CLIA) method. Quality control validation was performed for each batch to ensure all measured values fell within acceptable ranges.

### Establishment of biomarker reference intervals

In this study, the positivity of plasma biomarkers was determined based on clinical cut-off values established by Nanjing Vazyme Medical Technology Co., Ltd. These cut-offs were derived from EDTA plasma measurements of 1337 subjects (including 1139 healthy controls, 153 AD patients, and 45 non-AD neurological disease controls), optimized via receiver operating characteristic (ROC) curve analysis (maximizing Youden’s index) and validated through Bootstrap resampling (1000 iterations), with 95% confidence intervals varying by <5% across bootstrap samples. The specific criteria were as follows: Aβ42 positivity was defined as <4.70 pg/mL; Aβ42/Aβ40 positivity as <0.0560; p-tau181 positivity as >5.00 pg/mL; p-tau217 positivity as >5.00 pg/mL; GFAP positivity as >150 pg/mL; and NfL positivity as >125 pg/mL. Subjects not meeting these criteria were classified as negative.

### Cognitive function impairment assessment

When patients were clinically stable and cooperative, the MMSE was administered to assess cognitive function. The MMSE evaluates orientation, recall, memory, attention, calculation, language, and visuospatial abilities, with a total score of 30. A score below 27 indicates cognitive dysfunction, with lower scores correlating with more severe cognitive impairment [9].

### 18F-AV45 PET-CT image analysis and positivity assessment

This study included 18 patients who underwent 18F-AV45 PET/CT imaging using a General Electric Discovery MI Gen 2 PET/CT scanner. Subjects received an intravenous injection of florbetapir F18 at 0.13–0.15 mCi/kg based on body weight, followed by a 50-minute resting period before head PET/CT scanning. The scanning parameters included 120 kV voltage, 100 mA current, helical scanning with a 1.0 mm slice thickness and 0.8 pitch, and a 20-minute static PET scan in three-dimensional mode with a 336×336 matrix, covering the cranial vertex to the skull base. The images were analyzed by two experienced nuclear medicine physicians using interpretation criteria that entailed visual assessment of six Aβ-prone brain regions (frontal, temporal, parietal, occipital lobes, posterior cingulate cortex, and precuneus). Significant tracer uptake, increased cortical uptake, or blurring/disappearance of gyral-sulcal patterns indicated visual positivity, supplemented by semi-quantitative analysis calculating standardized uptake value ratios (SUVR) using the cerebellum as a reference region. A SUVR >1.11 in any target region indicated semi-quantitative positivity. Overall positivity was determined only when both visual and semi-quantitative assessments were positive, with negativity requiring both to be negative. Discrepant results necessitated joint review and consensus discussion.

### Blinding procedure

To ensure the objectivity and independence of all critical research components, this study implements a full-process blinded design. First, plasma biomarker detection employs a single-blind operation; all samples are anonymized and analyzed by laboratory personnel unaware of the clinical information. Second, PET-CT image analysis utilizes independent double-blind reading conducted by two nuclear medicine physicians who are unaware of patient details. They perform visual assessments and semi-quantitative analyses separately; if results differ, a joint review and consensus discussion is held. Neuropsychological assessments and clinical diagnoses are also executed independently, with evaluators lacking access to imaging or plasma data. Finally, after all data are aggregated and anonymized, researchers maintain blinding regarding participants’ clinical groupings during statistical analysis to systematically control potential bias and enhance the reliability of study outcomes.

### Ethical statement

This study received approval from the Ethics Committee of the General Hospital of Ningxia Medical University (Ethics Approval Number: KYLL-2025-1000).

### Statistical analysis

Data analysis was conducted using SPSS 27.0 and GraphPad Prism 10. Categorical data are presented as counts (percentages), while continuous variables are expressed as medians (interquartile ranges), given the non-normal distribution of most biomarkers. Normality and homogeneity of variance were assessed using the Shapiro-Wilk and Levene’s tests. Due to the departure of most plasma biomarkers from normality, non-parametric methods were employed. Group differences among HC, MCI, and AD were evaluated using the Kruskal-Wallis test, followed by Mann-Whitney *U* tests with Bonferroni adjustments for significant findings. Effect sizes (epsilon squared for the Kruskal-Wallis test; “r(U)” ═ |Z|/√N for pairwise comparisons) and 95% confidence intervals for median differences (Hodges-Lehmann estimator) are reported alongside “p” values. Statistical significance was defined as “p” < 0.05 (two-tailed). ROC curves were generated for seven indicators, and the area under the curve (AUC) was calculated to assess the diagnostic value of plasma biomarkers for MCI and AD. Monotonic associations between the seven plasma biomarkers and MMSE scores were evaluated using Spearman’s rank correlation analysis, with results reported as correlation coefficients (“rs”) and their corresponding 95% confidence intervals (CIs). A two-sided *p* value < 0.05 was considered statistically significant.

The consistency between the seven indicators and 18F-AV45 PET-CT imaging for detecting Aβ pathology was assessed by calculating Cohen’s Kappa coefficient.

## Results

### Participant characteristics

As detailed in Table 1, seventy participants aged 46–88 years were enrolled in this study. No significant age differences were found between the HC vs. MCI or HC vs. AD groups; however, MCI participants were significantly younger than those with AD (*P* < 0.01). This finding is consistent with the clinical understanding that MCI is typically identified at a younger age than AD. No statistically significant differences in gender distribution were observed across the groups. As anticipated, cognitive function, as measured by the MMSE, differed significantly among the three groups (*P* < 0.05). Post hoc pairwise comparisons indicated that both the MCI and AD groups had significantly lower MMSE scores compared to the HC group (*P* < 0.05 for both). Furthermore, the AD group exhibited significantly poorer cognitive performance than the MCI group (*P* < 0.01).

**Table 1:** Characteristics of participants

**Variables**	**Total (*n* ═ 70)**	**HC group** **(*n* ═ 15)**	**MCI group** **(*n* ═ 20)**	**AD group** **(*n* ═ 35)**	**Statistic**	***P***
Age, Mean ± SD	71.44 ± 8.24	72.60 ± 7.59	66.20 ± 7.53	73.94 ± 7.69	F = 6.79	0.002
Female, n (%)	43 (61.43)	9 (60.00)	12 (60.00)	22 (62.86)	χ^2^ ═ 0.06	0.970
MMSE	22 (15, 28)	29 (28, 30)	22 (20, 27)	16 (11, 22)	H = 34.84	<0.0001

This study did not comprehensively collect neuropsychiatric inventory scores beyond the MMSE. Furthermore, critical covariates, such as CSF biomarkers and APOE ɛ4 allele carrier status, were not systematically available for analysis and, consequently, are not included in this baseline comparison. Abbreviations: F: Analysis of variance (ANOVA); χ^2^: Chi-square test; SD: Standard deviation; H: Kruskal–Wallis H test; HC: Healthy controls; MCI: Mild cognitive impairment; AD: Alzheimer’s disease; MMSE: Mini-Mental State Examination; NPI: Neuropsychiatric Inventory; CSF: Cerebrospinal fluid; APOE: Apolipoprotein E.

### Comparison of plasma marker levels among the three groups

Kruskal-Wallis tests revealed that, with the exception of Aβ40, all six biomarkers exhibited significant differences among the three groups (all *P* ≤ 0.0013), with generally large overall effect sizes (ɛ^2^ ═ 0.192–0.415). Among these biomarkers, p-tau217 exhibited the largest between-group effect (ɛ^2^ ═ 0.415), followed by p-tau181 (ɛ^2^ ═ 0.389) and GFAP (ɛ^2^ ═ 0.318). Notably, all biomarkers that displayed significant overall differences also demonstrated significant pairwise differences between AD and HC groups, as well as between AD and MCI groups, while no significant differences were detected between the MCI and HC groups (all *P* > 0.05).

**Figure 1.**
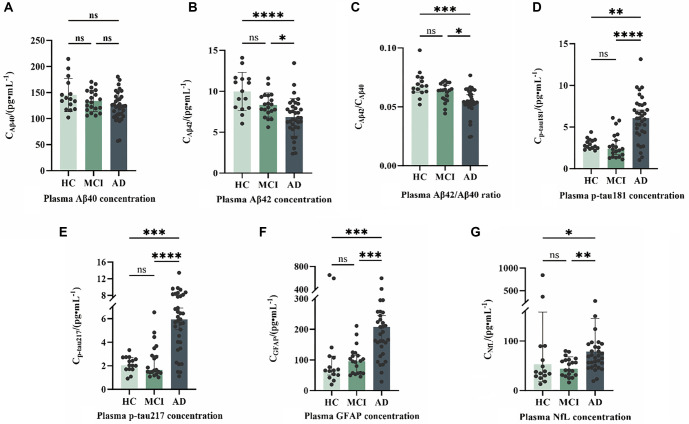
**Plasma biomarker concentrations across clinical groups.** Plasma levels of (A) Aβ40, (B) Aβ42, (C) Aβ42/Aβ40 ratio, (D) p-tau181, (E) p-tau217, (F) GFAP, and (G) NfL were compared among cognitively healthy controls (HC, *n* ═ 15), mild cognitive impairment (MCI, *n* ═ 20), and Alzheimer’s disease dementia (AD, *n* ═ 35). Dots represent individual participants; bars depict group central tendency with dispersion (median with interquartile range). Overall between-group differences were tested using the Kruskal–Wallis test, followed by pairwise Mann–Whitney *U* tests with Bonferroni adjustment. Horizontal brackets indicate pairwise comparisons. *ns*, not significant; *P* < 0.05; *P < 0.01; **P < 0.001; ***P < 0.0001. Broken y-axes in panels E–G indicate scale discontinuities introduced to accommodate high values/outliers while preserving readability of the main data distribution. Abbreviations: Aβ40: Amyloid-β40; Aβ42: Amyloid-β42; Aβ42/Aβ40: Amyloid-β42/Amyloid-β40 ratio; p-tau181: Phosphorylated tau 181; p-tau217: Phosphorylated tau 217; GFAP: Glial fibrillary acidic protein; NfL: Neurofilament light chain; HC: Healthy controls; MCI: Mild cognitive impairment; AD: Alzheimer’s disease; IQR: Interquartile range; ns: Not significant.

Amyloid-related biomarkers and tau protein biomarkers exhibited opposite directional changes in concentration, both demonstrating large to very large effect sizes. AD patients had Aβ42 levels that were, on average, 3.19 pg/mL lower than the HC group (95% CI [1.60, 4.74], r(U) ═ 0.543, *P* < 0.001), and the Aβ42/Aβ40 ratio was lower by 0.012 (95% CI [0.007, 0.017], r(U) ═ 0.545). Conversely, AD patients had p-tau217 levels that were 3.89 pg/mL higher than the HC group (95% CI [2.63, 5.11], r(U) ═ 0.606), and p-tau181 levels were higher by 3.19 pg/mL (95% CI [2.01, 4.23], r(U) ═ 0.567). All these effect sizes are classified as large to very large (r(U) > 0.475). The glial activation marker GFAP was significantly elevated in the AD group, with an increase of 110.86 pg/mL compared to the HC group (95% CI [51.18, 165.48], r(U) ═ 0.475). The axonal injury marker NfL also showed an increase in the AD group, although with relatively smaller effect sizes (AD vs. HC: r(U) ═ 0.337; AD vs. MCI: r(U) ═ 0.465). Interestingly, the effect size for GFAP was larger in the MCI–AD comparison (r(U) ═ 0.566) compared to the HC–AD comparison (r(U) ═ 0.475), and NfL exhibited a similar pattern, suggesting that these markers undergo continuous changes during disease progression (Figure 1).

### Diagnostic accuracy of plasma biomarkers

ROC curves were utilized to evaluate the efficacy of the seven plasma biomarkers in diagnosing AD and differentiating MCI from AD.

**Figure 2.**
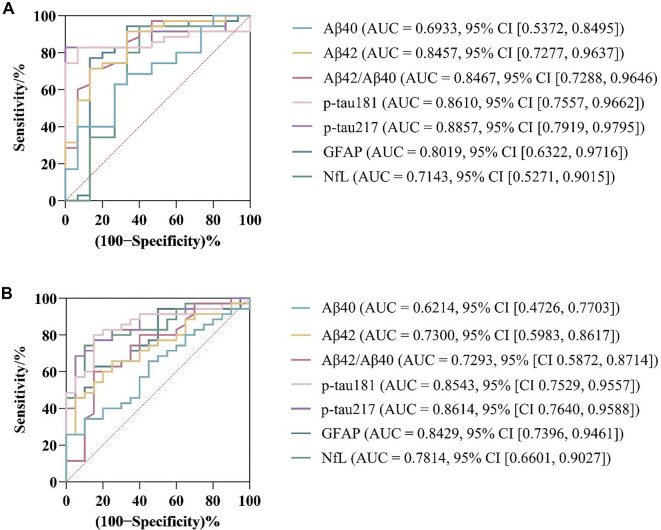
**Diagnostic accuracy of plasma biomarkers assessed by receiver operating characteristic (ROC) analysis.** (A) ROC curves for plasma Aβ40, Aβ42, Aβ42/Aβ40 ratio, p-tau181, p-tau217, GFAP, and NfL in discriminating participants with Alzheimer’s disease dementia (AD; *n* ═ 35) from cognitively healthy controls (HC; *n* ═ 15). (B) ROC curves for discriminating mild cognitive impairment (MCI; *n* ═ 20) from AD (*n* ═ 35) using the same biomarker panel. Sensitivity is plotted against 100–specificity; the diagonal reference line denotes no discriminative ability (AUC = 0.50). For each biomarker, the AUC and its 95% CI are annotated on the plot. Abbreviations: ROC: Receiver operating characteristic; AUC: Area under the curve; CI: Confidence interval; Aβ40: Amyloid-β40; Aβ42: Amyloid-β42; Aβ42/Aβ40: Amyloid-β42/Amyloid-β40 ratio; p-tau181: Phosphorylated tau 181; p-tau217: Phosphorylated tau 217; GFAP: Glial fibrillary acidic protein; NfL: Neurofilament light chain; HC: Healthy controls; MCI: Mild cognitive impairment; AD: Alzheimer’s disease.

In diagnosing AD (Figure 2A), p-tau217 exhibited the highest AUC of 0.8857 (95% CI [0.7919, 0.9795]), followed by p-tau181 (AUC = 0.8610, 95% CI [0.7557, 0.9662]), the Aβ42/Aβ40 ratio (AUC = 0.8467, 95% CI [0.7288, 0.9646]), and Aβ42 (AUC = 0.8457, 95% CI [0.7277, 0.9637]). GFAP, NfL, and Aβ40 demonstrated moderate diagnostic performance, with AUCs of 0.8019 (95% CI [0.6322, 0.9716]), 0.7143 (95% CI [0.5271, 0.9015]), and 0.6933 (95% CI [0.5372, 0.8495]), respectively.

**Figure 3.**
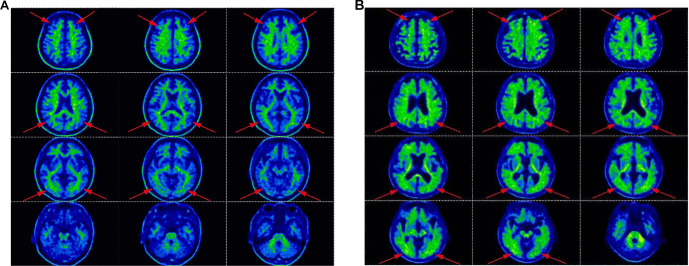
**Representative ^1^^8^F-florbetapir (^1^^8^F-AV45) amyloid PET brain images illustrating negative and positive amyloid status.** (A) Amyloid-negative scan showing low cortical tracer binding with preserved gray–white matter differentiation (cortical gray matter uptake lower than adjacent white matter). (B) Amyloid-positive scan demonstrating increased, diffuse cortical tracer uptake with blurring or loss of the normal gray–white matter contrast, consistent with cerebral β-amyloid deposition. Red arrows highlight cortical regions used for visual interpretation (e.g., frontal, parietal, temporal, and posterior cingulate/precuneus areas) in accordance with study reading criteria. Abbreviations: ^1^^8^F-AV45: ^1^^8^F-florbetapir; PET: Positron emission tomography.

For differentiating MCI from AD (Figure 2B), p-tau217 again exhibited the highest accuracy (AUC = 0.8614, 95% CI [0.7640, 0.9588]), closely followed by p-tau181 (AUC = 0.8543, 95% CI [0.7529, 0.9557]) and GFAP (AUC = 0.8429, 95% CI [0.7396, 0.9461]). NfL demonstrated an AUC of 0.7814 (95% CI [0.6601, 0.9027]). The Aβ markers (Aβ42, Aβ42/Aβ40 ratio, and Aβ40) exhibited lower discriminatory power, with AUCs of 0.7300 (95% CI [0.5983, 0.8617]), 0.7293 (95% CI [0.5872, 0.8714]), and 0.6214 (95% CI [0.4726, 0.7703]), respectively.

### Correlation between plasma biomarker levels and MMSE scores

Plasma Aβ42 and the Aβ42/Aβ40 ratio displayed significant positive correlations with MMSE scores (Aβ42: rs = 0.266, 95% CI [-0.007, 0.502], *P* ═ 0.049; Aβ42/Aβ40: rs = 0.472, 95% CI [0.229, 0.660], *P* ═ 0.0003). In contrast, plasma levels of p-tau181, p-tau217, GFAP, and NfL showed significant negative correlations with MMSE scores (p-tau181: rs ═ –0.426, 95% CI [--0.626, --0.173], *P* ═ 0.0012; p-tau217: rs ═ --0.456, 95% CI [--0.648, --0.209], *P* ═ 0.0005; GFAP: rs ═ --0.415, 95% CI [--0.618, --0.161], *p* ═ 0.0016; NfL: rs ═ --0.408, 95% CI [--0.613, --0.152], *P* ═ 0.0020). No significant correlation was found between plasma Aβ40 and MMSE scores (rs = 0.017, 95% CI [--0.257, 0.289], *P* ═ 0.901).

### Correlation between plasma marker levels and 18F-AV45 PET-CT visualization

Eighteen patients underwent 18F-AV45 PET-CT, including 4 patients with MCI and 14 patients with AD. Aβ deposition was absent in all 4 MCI patients but present in all 14 AD patients. Cohen’s Kappa analysis indicated substantial agreement for plasma p-tau181 (Kappa = 0.727, 95% CI [0.384, 1.000], s*p* ═ 0.001). Moderate agreement was observed for the plasma Aβ42/Aβ40 ratio and p-tau217 (Kappa = 0.526, 95% CI [0.165, 0.887], *p* ═ 0.011) and for plasma GFAP (Kappa = 0.557, 95% CI [0.116, 0.998], *P* ═ 0.017). Plasma NfL exhibited negligible and statistically non-significant agreement (Kappa = 0.033, 95% CI [--0.038, 0.104], *P* ═ 0.582). Due to a lack of variance in binary classification, plasma Aβ42 could not be meaningfully assessed for agreement (Figure 3).

## Discussion

This study utilized acridinium ester-based direct chemiluminescence for the analysis of plasma samples for Aβ40, Aβ42, Aβ42/Aβ40, p-tau181, p-tau217, GFAP, and NfL in the HC, MCI, and AD dementia groups. Our findings demonstrated significant differences between AD patients and those with HC and MCI. Notably, plasma p-tau181 and p-tau217 exhibited the highest diagnostic performance, with AUC values of 0.8610 and 0.8857, respectively, in distinguishing AD dementia patients from healthy individuals, corroborating previous studies [10, 11]. These data suggest that these biomarkers possess substantial clinical diagnostic value in a cross-sectional context, indicating their potential as core biomarkers for AD diagnosis. Brickman et al. [12] reported that among available plasma biomarkers, p-tau217 concentrations reflect underlying tau pathology with the greatest fidelity and assist in the clinical diagnosis of AD. Furthermore, results from Palmqvist et al. [13] indicated that p-tau217 surpassed p-tau181 in pathological assays in neuropathology. Our findings regarding p-tau217 align with those of Palmqvist et al.; however, we did not observe a significant difference between p-tau217 and p-tau181, possibly due to the limited sample size of this study.

The predictive values of Aβ42 (AUC = 0.846) and the Aβ42/Aβ40 ratio (AUC = 0.847) for AD were significantly higher than those of Aβ40 alone (AUC = 0.693), further underscoring the importance of Aβ42 deposition in AD amyloid pathology [14]. AD is pathologically characterized by widespread neuronal loss and the presence of two hallmark protein deposits in the brain: extracellular amyloid plaques and intracellular neurofibrillary tangles (NFTs), resulting from the hyperphosphorylation of tau protein [15]. Plasma levels of Aβ42 and the Aβ42/Aβ40 ratio serve as valuable indicators for assessing amyloid plaque formation in the brain. Elevated Aβ accumulation contributes to brain toxicity, potentially leading to significant cognitive impairment or decline [16], which is reflected peripherally through the sequestration or depletion of Aβ in bodily fluids, including blood. Our findings of reduced levels of Aβ42 and the Aβ42/Aβ40 ratio align with the hypothesis that increased Aβ deposition in the brain corresponds to a depletion of peripheral Aβ42 and the Aβ42/Aβ40 ratio.

Both p-tau181 and p-tau217 levels were significantly elevated in AD patients compared to those with MCI, with both biomarkers demonstrating similar efficacy in distinguishing AD from MCI (AUC: 0.8543 and 0.8614, respectively). Conversely, neither biomarker was elevated in the MCI group relative to cognitively normal HC group, indicating their limited ability to differentiate MCI from HC. Previous studies have similarly shown that without considering Aβ pathological changes, plasma biomarkers lack significant effectiveness in distinguishing MCI from HC across diverse geographical and ethnic populations [17]. It is evident that the MCI patient group—being the most probable target for early intervention—requires more sensitive assays for detection.

To investigate the association between plasma biomarkers and cognitive status, we found that plasma Aβ42 and the Aβ42/Aβ40 ratio exhibited significant positive correlations with MMSE scores in patients with MCI and AD. In contrast, plasma levels of p-tau181, p-tau217, GFAP, and NfL showed significant negative correlations with MMSE scores. These results indicate that lower levels of Aβ42 and the Aβ42/Aβ40 ratio, alongside higher levels of p-tau181, p-tau217, GFAP, and NfL, are associated with more severe cognitive impairment. Notably, p-tau217 demonstrates diagnostic performance comparable to cerebrospinal fluid-based measures [10]. A longitudinal study on AD biomarkers revealed that the most rapid changes in biomarker concentrations occurred in individuals with MMSE scores between 25 and 27, suggesting that significant biomarker changes manifest during the early stages of cognitive decline [18]. Therefore, integrating MMSE scores with biomarker levels may aid in determining the optimal timing for clinical intervention. While GFAP and NfL serve as secondary markers to core AD biomarkers like Aβ and tau, they provide complementary insights regarding astroglial activation and neuronal injury, respectively. Their dynamic changes across disease stages reinforce the notion that multiple pathological mechanisms—neuroinflammation and axonal degeneration—co-occur and can be tracked through plasma to offer a comprehensive view of AD progression [19].

Using ante-mortem florbetapir F18 PET-CT imaging in clinical patients, Carome et al. [20] successfully predicted the presence of Aβ deposition in the brain during post-mortem autopsy. Thus, molecular imaging techniques can identify Aβ pathology in the brain during an individual’s lifetime. Aβ deposition was predominantly observed in the temporal, frontal, occipital, posterior cingulate gyrus, and precuneus lobes. By correlating the positive status of each plasma marker individually with the presence or absence of brain amyloid deposits observed in the 18F-AV45 PET-CT examination, we established a relationship between blood tau protein levels and Aβ deposition in the brain. This cross-sectional analysis revealed moderate to good agreement between plasma biomarkers (Aβ42/Aβ40 ratio, p-tau181, and p-tau217) and Aβ-PET status, indicating that a lower Aβ42/Aβ40 ratio and higher levels of p-tau181 and p-tau217 are associated with PET-positive Aβ deposition. Among these, p-tau181 demonstrated the strongest correlation, followed by the Aβ42/Aβ40 ratio and p-tau217, while plasma NfL showed no significant agreement. The Aβ42/Aβ40 ratio is commonly employed to reduce inter-individual variability in blood-based assays and provides a more consistent reflection of cerebral Aβ pathology than Aβ42 or Aβ40 alone [21]. In contrast, plasma p-tau markers, particularly p-tau181 and p-tau217, may provide greater diagnostic value than amyloid measures alone, as indicated by their ability to differentiate cases based on amyloid-PET or tau-PET results in previous studies [11].

This cross-sectional study demonstrates distinct plasma biomarker profiles across clinical groups, with AD patients showing pronounced alterations in amyloid, tau, glial, and axonal markers compared to both MCI and HC groups. Notably, p-tau217 and p-tau181 exhibited the strongest between-group discrimination, supported by large effect sizes and high diagnostic accuracy (AUC = 0.8857 for AD vs. HC; 0.8614 for AD vs. MCI). In contrast, no biomarker reliably distinguished MCI from healthy controls, highlighting the ongoing challenge of early detection at the MCI stage. Furthermore, our findings suggest that the plasma Aβ42/Aβ40 ratio, p-tau181, and p-tau217 show exploratory consistency with Aβ deposition as detected by 18F-AV45 PET-CT, particularly in the subgroup with a small sample size. Collectively, these results emphasize the clinical potential of plasma biomarkers—especially p-tau181, p-tau217, and the Aβ42/Aβ40 ratio—in supporting AD diagnosis within a cross-sectional framework, while also underscoring the need for further validation in larger, longitudinal cohorts to better understand their longitudinal trajectories and clinical associations.

Importantly, unlike many current studies that rely on highly specialized platforms (e.g., Simoa), we innovatively assessed these core AD biomarkers using a fully automated chemiluminescence immunoassay—a technology widely available in routine clinical laboratories. This represents a significant advancement toward practical clinical translation rather than a technological compromise. Future longitudinal and large-scale studies are needed to further validate the generalizability and diagnostic performance of this accessible approach.

This study has several limitations. First, the findings are limited by the relatively small sample size and cross-sectional design. The low number of PET-negative cases (*n* ═ 4) results in wide confidence intervals for agreement estimates, diminishing their precision, while the absence of longitudinal data precludes any assessment of biomarker dynamics or predictive utility for disease progression. Second, the single-center, case-control design may introduce spectrum and selection biases, as the AD and MCI groups consisted of clinically referred patients, whereas the HC group included community volunteers. This design could lead to an overestimation of biomarker differences between groups, given that the patient sample may represent a more clinically pronounced or diagnostically clearer spectrum of disease. Third, cognitive assessment relied solely on the MMSE, a global screening tool that lacks sensitivity to domain-specific deficits characteristic of AD; future studies would benefit from incorporating comprehensive neuropsychological assessments. Lastly, the unavailability of data on key covariates (e.g., detailed vascular risk factors, medication history, and APOE ɛ4 genotype) and the use of a clinically accessible but novel chemiluminescence immunoassay platform—which requires further cross-platform validation—also warrant consideration. Future multi-center studies with larger, prospectively followed cohorts, more detailed phenotypic characterization, and adjustment for potential confounders are necessary to confirm and extend these exploratory findings.

## Conclusion

Direct measurements of plasma biomarkers using chemiluminescence immunoassay—particularly p-tau181, p-tau217, and the Aβ42/Aβ40 ratio—provide substantial clinical value for AD diagnosis in a cross-sectional context.

## Data Availability

Due to privacy protection, the data provided in this study are available upon request from the corresponding author.
